# Complexity Measures of Voice Recordings as a Discriminative Tool for Parkinson’s Disease

**DOI:** 10.3390/bios10010001

**Published:** 2019-12-20

**Authors:** Rekha Viswanathan, Sridhar P. Arjunan, Adrian Bingham, Beth Jelfs, Peter Kempster, Sanjay Raghav, Dinesh K. Kumar

**Affiliations:** 1 School of Engineering, RMIT University, Melbourne VIC 3000, Australia; pvrekha.777@gmail.com (R.V.); sanjay.raghav@rmit.edu.au (S.R.); dinesh@rmit.edu.au (D.K.K.); 2 Department of Electronics and Instrumentation Engineering, SRM Institute of Science and Technology, Chennai 603203, India; 3 Department of Neurology, Monash Medical Center, Melbourne VIC 3168, Australia; Peter.Kempster@monashhealth.org

**Keywords:** complexity, fractal dimension, normalised mutual information, Parkinson’s disease, sustained phonemes, dysarthria

## Abstract

In this paper, we have investigated the differences in the voices of Parkinson’s disease (PD) and age-matched control (CO) subjects when uttering three phonemes using two complexity measures: fractal dimension (FD) and normalised mutual information (NMI). Three sustained phonetic voice recordings, /a/, /u/ and /m/, from 22 CO (mean age = 66.91) and 24 PD (mean age = 71.83) participants were analysed. FD was first computed for PD and CO voice recordings, followed by the computation of NMI between the test groups: PD–CO, PD–PD and CO–CO. Four features reported in the literature—normalised pitch period entropy (Norm. PPE), glottal-to-noise excitation ratio (GNE), detrended fluctuation analysis (DFA) and glottal closing quotient (ClQ)—were also computed for comparison with the proposed complexity measures. The statistical significance of the features was tested using a one-way ANOVA test. Support vector machine (SVM) with a linear kernel was used to classify the test groups, using a leave-one-out validation method. The results showed that PD voice recordings had lower FD compared to CO (*p* < 0.008). It was also observed that the average NMI between CO voice recordings was significantly lower compared with the CO–PD and PD–PD groups (*p* < 0.036) for the three phonetic sounds. The average NMI and FD demonstrated higher accuracy (>80%) in differentiating the test groups compared with other speech feature-based classifications. This study has demonstrated that the voices of PD patients has reduced FD, and NMI between voice recordings of PD–CO and PD–PD is higher compared with CO–CO. This suggests that the use of NMI obtained from the sample voice, when paired with known groups of CO and PD, can be used to identify PD voices. These findings could have applications for population screening.

## 1. Introduction

Parkinson’s disease (PD) is a progressive neurological condition. Its motor disabilities are caused by a degeneration of the dopamine-releasing nerve cells in the substantia nigra. Other neuronal systems are also affected, and this contributes to the various nonmotor features of the disorder [1]. One challenge in the search for disease-modifying treatments is the difficulty in identifying PD at an early stage, before its motor signs are clearly developed. Later, fluctuating responses to symptomatic treatment make it hard to monitor the progression of PD [2,3]. Early detection and monitoring of progression using objective measures can play an important role in maintaining quality of life in PD by facilitating symptomatic medical interventions [4,5]. There are a limited number of motor features that have been identified as appearing in the early stages of PD, often before clinical diagnosis [3,6,7]. As such, measurements of changes in gait [8,9], handwriting [10,11] and speech [12,13] have been proposed for the early, computer-assisted diagnosis of the disease.

Dysarthria [14] is commonly observed in PD patients [12,13], and is often one of the first symptoms. It results from a combination of impairments to upper airway, laryngeal and respiratory muscle control. The non-invasive nature of speech analysis makes it an attractive tool to aid in the prediction or early detection of PD. Using automated speech analysis to diagnose and monitor PD has several advantages, primary among them the ability to make unbiased and objective measurements. Importantly, speech analysis also allows the progression of the disease to be monitored remotely [15,16], reducing the number and expense of clinical visits. Acoustic analysis of the voice of PD patients also has therapeutic applications. Voice analysis can be used when training patients to improve their voice. The Lee Silverman voice treatment (LSVT) is one such system that combines voice analysis and feedback to patients [17].

Studies have reported the use of acoustic analysis to identify vocal pathologies in PD [18,19,20]. The voice of PD patients is associated with low volume, monotonicity, high pitch, tremulousness, breathiness, hoarseness and, sometimes, the inability to spontaneously maintain loudness [21]. Perturbation measures are the most commonly used acoustic features; these determine the intercycle differences in frequency and amplitude of the speech signal [22,23]. However, it has been shown that perturbation measures, especially jitter and shimmer, can be inconsistent when applied to aperiodic signals [24,25] and may give invalid results when differentiating pathological from normal voices [26]. Recent advances in speech analysis for the diagnosis of PD have shown that nonlinear features such as detrended fluctuation analysis (DFA), correlation dimension (D2), Lyapunov exponent (L1) and recurrence pitch period entropy (RPDE) are more suitable for distinguishing between the voices of controls (CO) and PD patients [27,28,29]. These features may discern PD from CO subjects based on the presence of aperiodicity in the vocal fold vibration during sustained phonation, which correlates with a perceptually rough or hoarse voice [30,31]. However, with nonpathologically large differences in the voices of people in any population, detecting the differences between PD and CO subjects at early stages of PD can be difficult.

Speech contains complex information that is embedded in the acoustic features [32]. This information includes, but is not restricted to, markers of sex, age, health, psychological state, voice production mechanism and linguistic mechanisms [32]. Healthy people can modify their voices to generate different sounds that require fine control and coordination of the articulatory and respiratory muscles. However, PD patients have reduced neuromotor control, which affects the voice mechanism and thus the sounds produced. Machine analysis of speech could augment standard clinical assessments [33] in the early detection of PD, but there are significant inter- and intra-individual variations [32], which makes this challenging. Thus, there is a need to find features for identifying differences between healthy and pathological voices that can be adapted for a given population.

It is well recognised from the literature that aging and disease change the complexity of biological systems [34,35,36,37]. Based on the above and on evidence from other nonspeech PD studies [38,39], we hypothesize (Hypothesis 1) that the voice of PD patients will have reduced complexity when compared with CO, indicative of reduced vocal dynamics (richness). We also hypothesize (Hypothesis 2) that, because of the reduced complexity in the voice of PD patients, the information shared between them will be greater than the information shared between voices of CO. The shared information can be in terms of the temporal and spatial information in the voice recordings, which reflect periodicity, fine structures and fundamental frequency. We use two complexity measures—fractal dimension (FD) and normalised mutual information (NMI)—to test these hypotheses.

Fractals can be described as infinitely complex self-similar patterns across different scales and are created by a simple process continually reiterated in a feedback loop [35]. Fractal dimension (FD) is generally employed to measure the complexity of such fractal objects. While measured in the temporal domain, FD provides an estimation of the overall complexity in the signal and has been used in biomedical applications to quantify complexity in biosignals [40,41].

Mutual information (MI) is a measure of the dependence between the two time series and includes both linear and nonlinear relationships [34]. It is estimated based on the independent entropies and joint entropy of the time series; MI has a lower bound of zero and the upper bound of MI is determined by the independent entropies [42,43]. Difficulties arise when comparing data, but they can be minimised by normalising the MI, termed Normalised Mutual Information (NMI). One application of MI is feature selection, which has been the focus of many recent dysphonic speech studies [35,36,37]. In these, phonetic recordings were analysed to obtain all potential features, from which the most suitable ones were selected. There are several advantages to computing NMI directly from the raw phonetic sound recordings that address some of the limitations of existing acoustic features. While a lot of speech features require accurate detection of the fundamental frequency or pitch of the speech signal [44], by applying the calculation directly to the time series, NMI provides quantification of the dependency between signals.

In this study, Hypothesis 1 was verified using FD, and Hypothesis 2 with NMI. This paper is organized as follows: Section 2 provides a brief introduction to FD, NMI, the experimental protocol and the data analysis methods. Results are presented in Section 3, with a discussion provided in Section 4. Finally, Section 5 concludes the paper.

## 2. Materials and Methods

### 2.1. Participants

Twenty-four patients diagnosed with PD within the last 10 years were recruited from the Movement Disorders Clinic at Monash Medical Centre. All complied with the Queen Square Brain Bank criteria for idiopathic PD [45]. Twenty-two healthy, similarly aged controls were also recruited. The presence of any advanced PD clinical milestone—visual hallucinations, frequent falling, cognitive disability or need for institutional care—was an exclusion criterion [46]. The study protocol was approved by the ethics committee of Monash Health, Melbourne, Australia (LNR/16/MonH/319) and RMIT University Human Research Ethics Committee, Melbourne, Australia (BSEHAPP22-15KUMAR). Written informed consent was obtained from all the participants before the experiments. Besides the recording of their voice, we also collected information regarding patient demographics, medication and health history. The motor section of UPDRS-III and the Montreal Cognitive Assessment (MoCA) were assessed and recorded.

Table 1 shows the patient information and demographics. The UPDRS-III score [33] of all participants shows clear differences between the groups, with mean scores of PD = 27.58 and CO = 2.64. The UPDRS-III motor assessment evaluates the overall deterioration of motor skills using 15 items, which include speech evaluation. The speech is rated between 0 and 4, where 0 corresponds to normal speech and 4 signifies unintelligible speech. In our study, the speech assessment for UPDRS-III showed that all CO had normal speech (score 0), which indicated that they did not have any underlying speech problems. PD patients in this study showed speech variation between normal (score 0) and minor loss of expression, diction and volume in their speech (score 1).

### 2.2. Data Recording

The sustained vowel sounds /a/ and /u/ have already been used in differentiating PD from CO [47,48,49]. However, vowel sounds are less dependent on active articulators. Earlier studies have reported the use of consonants like /b/, /d/ and /t/, which were found to provide the highest difference between the two groups [50,51]. Our previous investigative study has shown that the consonant /m/ provides better differentiation between PD and CO [52]. In this study, for the purpose of comparison with other studies, three different sustained phonemes have been investigated: /a/, /u/ and /m/.

Voice recordings of sustained vowel sounds /a/ (as in car), /u/ (as in wool) and a sustained consonant phonation /m/ (as in ham) were recorded from 24 PD and 22 CO participants. The phonetic sound recordings were collected in the off state of medication for the PD participants. Off state was defined as fasting, with anti-Parkinsonian medication withheld for at least 12 h.

Phonetic sound recordings were collected using a smartphone with a sampling rate of 48 kHz and 16-bit resolution [53,54] using an omnidirectional head-worn microphone; the recordings were saved using the WAV format. The recordings were collected from Monash Health Medical Centre, Melbourne, Australia. The data for the study were recorded in a noise-restricted room. Each recording contained only a single sustained phoneme utterance that was no longer than 30 s. As described, three phonemes were recorded from each participant, with 60 s to relax between each recording.

### 2.3. Computation of Complexity Measure

FD is a non-negative real value that quantifies the irregularity and complexity within the signal. FD has previously been reported for speech analysis and identifying speech pathology [55,56,57]. The FD of a signal can be estimated using two approaches: time domain and phase space methods. In the time domain, FD is estimated directly on the signal, and in the phase space method the FD of an attractor in phase space is estimated. Estimating FD in the time domain is computationally less complex. There are several algorithms for the computation of FD in time domain such as Higuchi’s algorithm [58], Katz’s algorithm [59] and Petrosian’s algorithm [60]. In this study, FD was computed using the box-counting method to measure the complexity in the time domain. The estimation of FD was performed using the FracLab [61] toolbox in MATLAB, Natick, MA, USA. A reduction in FD can be considered as a reduction in complexity of the phonetic recording [62]. The FD using the box counting method is given by the following equation:

(1) $$\mathrm{FD}=\;\underset{\epsilon \to 0}{\lim}\frac{\ln N\left(\epsilon \right)}{\ln\left(\epsilon \right)},$$

where N(ϵ)N(ϵ) is the minimum number of *n* dimensional boxes and ϵϵ is the side length of the boxes.

### 2.4. Normalised Mutual Information

Mutual Information (MI) is a measure to estimate the amount of shared information between the two random variables, in this case they are the phonetic sound recordings of CO and PD participants. MI is estimated based on the probability distributions of the random variables [63] as follows:

(2) $$I\left(X;Y\right)=\underset{x\in X}{\sum }\underset{y\in Y}{\sum }P_{XY\;}\left(x,y\right)log_{n}\frac{P_{XY}\left(x,y\right)}{P_{X}\left(x\right)P_{y}\left(y\right)},$$

where *I (X;Y)* is the MI of two random variables *X* and *Y*, and *P_XY_*, *P_X_*, and *P_Y_* are the joint and marginal probability distributions of the variables. The units used to measure MI are dependent on the value of *n* in (1). In our study, the value of *n* was 2 and thus the unit of MI was in bits. MI can be computed based on the entropy and joint entropy of the random variables, where the entropy of a variable *X* is given by

(3) $$H\left(X\right)=-\underset{x\in X}{\sum }P_{X}\left(x\right)log_{n}P_{X}\left(x\right),$$

resulting in MI being defined as

**Figure 1:**
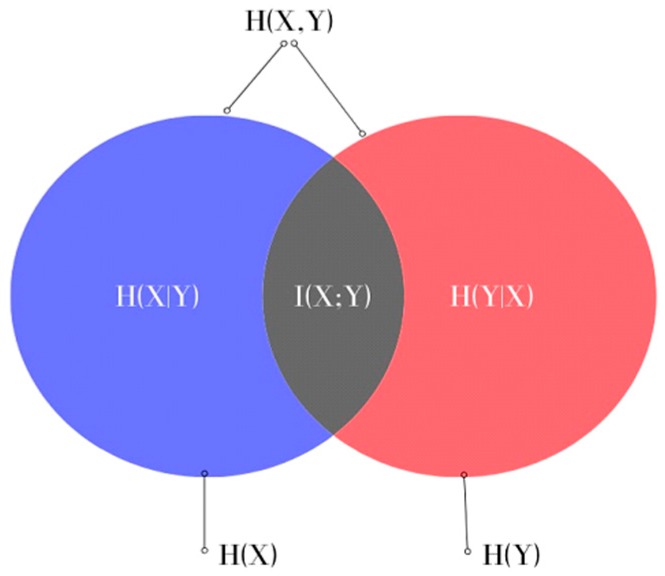
Venn diagram representing the relationship between MI and the entropies of *X* and *Y* in Equation (4).

*I*(*X*;*Y*) = *H*(*X*) + *H*(*Y*) − *H*(*X*,*Y*), (4)

where *H(X)* and *H(Y)* are the entropies of the random variables *X* and *Y*, which measure the uncertainty of *X* and *Y*, respectively, and *H(X,Y)* is the joint entropy measuring the uncertainty associated with variables *X* and *Y*. This can also be represented in the form of a Venn diagram, as shown in Figure 1.

MI has a lower bound of zero and the upper bound is determined by the information content of the variables [42,43]. For comparison, this needs to be normalised. The method [64] used to normalise the MI in this study is

(5) $$NMI\left(X;Y\right)=\frac{I\left(X;Y\right)}{\min\left(H\left(X\right),H\left(Y\right)\right)},$$

where *NMI (X;Y)* is the normalised mutual information of *X* and *Y* obtained by dividing the MI by the smallest of the entropies of *X* and *Y* and has a range between 0 and 1. High NMI indicates a higher proportion of shared information in the signals and also shows the signals to be more dependent. The NMI of a variable with itself is the maximum, 1, while a low NMI indicates a lower proportion of shared information in the signals and thus represents a lack of correlation between the signals.

In this study, we have followed the methodology proposed in [65] to determine the NMI. It was calculated based on the probability distributions of the phonetic sound recordings considered in pairs. The probability distributions of the recordings were calculated using the histogram method. The bins used in the histograms had uniform partitions and were determined using the Rice rule [66] equation for the number of bins:

(6) $$k=\lceil 2\sqrt[{3}]{N}\rceil ,$$

where *N* is the number of samples in the data and k is the number of bins.

### 2.5. NMI Computation

Before the computation of NMI, the sound recordings were pre-processed. This involved trimming the recordings by removing the unvoiced parts at the beginning and end, and then bandpass filtering the recordings between 80 Hz and 24 kHz. Since the NMI can only be evaluated between the time series of equal lengths, the first 2 s of all the recordings after pre-processing were used for analysis. The length of recordings was chosen to be 2 s since a substantial number of PD patients included in the study were not able to produce sustained phonation longer than that due to lack of lung capacity. To determine the histograms for the probability distributions, a window size of 480 samples was used, which was based on the Rice rule [66] and equates to using a histogram with 16 bins. Thus, the window size selected corresponds to 10 ms, which is one of the lengths recommended in the literature for analysing PD and healthy voices [67,68,69].

The methodology to evaluate the NMI for one phoneme is given below. If we consider two groups of sound recordings *A* and *B*, then to study the proportion of shared information between all of the sound recordings in these groups, the NMI was calculated pairwise as follows:

- A recording from group *A* is paired with a recording from group *B* and the NMI value computed;
- This is then repeated, pairing the recording from group *A* with each of the other recordings from group *B* in turn and calculating the NMI values;
- These values are then averaged to obtain a mean NMI value, which represents the average proportion of shared information between the recording in group *A* and all recordings from group *B*;
- This procedure is then repeated for all other voice recordings within group *A* to give a set of average values for group *A* paired with group *B* (*A*–*B* average NMI set).

In this study the NMI was first computed for the PD patients and CO subjects separately, giving the within-group sets of average NMI values:

1. *CO–CO*, where each recording from the CO group is paired with all other recordings from the CO group to obtain the average NMI values;
2. *PD–PD*, where each recording from the PD group is paired with all other recordings from the PD group to obtain the sets of average NMI values.

The next step was to evaluate the differences between the CO and the PD; the between-group sets of average NMI values were also calculated:

3. *CO–PD*, where each recording from the PD group is paired with all recordings in the CO group to obtain the sets of average NMI values.

This was repeated for all three phonetic signals collected from each study participant.

### 2.6. Computation of Comparative Speech Features

To provide a means of comparison with existing techniques, four features that have previously been reported to be successful at identifying pathological voices were also computed. These features were:

- Normalised pitch period entropy (Norm. PPE) [28,49] has been proposed to measure the lack of control in maintaining a stable pitch during sustained phonation. An increase in norm. PPE represents a deviation from healthy speech production [26,50];
- Glottal to noise excitation ratio (GNE) [51,52] measures the degree of turbulence in the voice by employing the method of inverse filtering of the vocal signal [51];
- Detrended fluctuation analysis (DFA) [28,53] measures the scaling exponent of the time series. It is a degree of the autocorrelation in the signal [54], and in speech analysis this corresponds to the presence of turbulent noise. Thus, the richness of speech should indicate a lower value of the scaling exponent;
- Glottal closing quotient (ClQ) [28] represents the time duration for which the glottis is closed [55]. This feature is primarily associated with different types of voice quality: breathy, modal and creaky. An increase in ClQ is observed as a breathy voice quality [56].

### 2.7. Statistical Analysis

The sets of average NMI values and FD estimated in this study and the features used for comparison extracted from the three phonetic sound recordings were tested and found to be normally distributed. The normality was tested using the Anderson–Darling test. Therefore, we used parametric statistics (one-way ANOVA) to examine the differences between the sets.

### 2.8. Classification Analysis

After computing FD and average NMI for the group sets, the receiver operating characteristics (ROC) wrtr generated and the area under the curve (AUC) was computed. This was also repeated for the comparison features mentioned in Section 2.6 that have been reported in the literature to differentiate PD and CO. The support vector machine (SVM) is a supervised machine learning technique that iteratively identifies a hyperplane that separates the two classes after they are transformed with the help of kernel functions [70]. An SVM classifier has been shown to be effective for classifying PD patients using speech features [70,71]. In this study, an SVM classifier with a linear kernel was employed with a leave-one-subject-out validation method and the false negatives, false positives, true positives and true negatives of the classification were measured.

## 3. Results

### 3.1. Statistical Analysis

#### 3.1.1. Complexity of the Phonetic Sound Recordings

**Figure 2:**
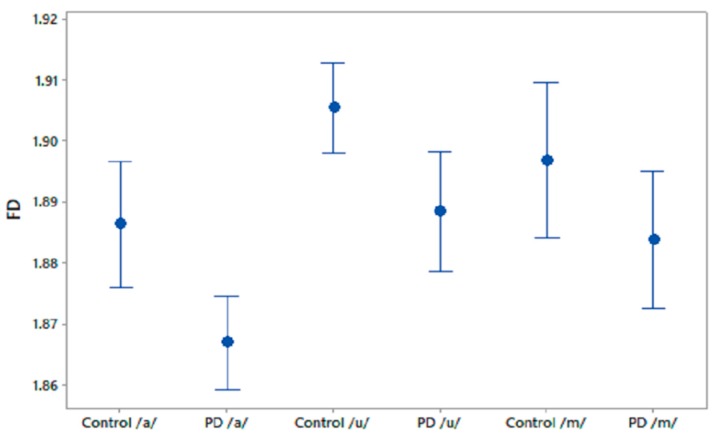
95% CI plots for the features FD for PD and CO.

**Figure 3:**
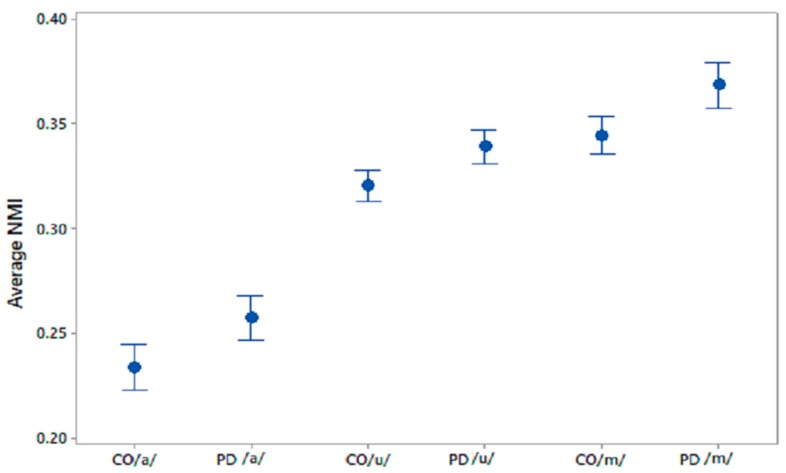
95% CI plots of average NMI values for CO–CO, PD–PD for /a/, /u/, /m/.

We computed FD for the phonetic sound recordings of PD patients and CO subjects. The FD values were compared using one-way ANOVA test and the results are shown in Table 2. It was observed that FD differentiated PD patients and CO subjects in recordings of /a/ and /u/. Figure 2 shows the 95% CI plots of FD for PD and CO subjects. Figure 3 shows higher FD in CO for the three phonetic recordings than for PD patients. Higher FD for CO subjects shows that the phonetic signals of CO are more complex than those of PD patients.

#### 3.1.2. Within-Group Average NMI

After establishing that PD has lower FD compared to CO, the next step was to compare the proportion of shared information within cohorts of PD and CO by calculating the within-group sets of NMI values [37] using the average NMI, and a comparison of the average NMI for CO–CO and PD–PD was made. The range of values in each set of average NMI is shown in Table 3 for the phonetic recordings of /a/, /u/, and /m/, respectively.

The differences in the within-group average NMI for PD and CO were statistically evaluated using one-way ANOVA; the results are shown in Table 4. The sets of average NMI values for CO–CO and PD–PD were significantly different for all phonemes with *p* values *<* 0.005. 95% CI plots for the average NMI for COs and PD patients are shown in Figure 3 for all three phonetic recordings.

#### 3.1.3. Between-Group Average NMI

**Figure 4:**
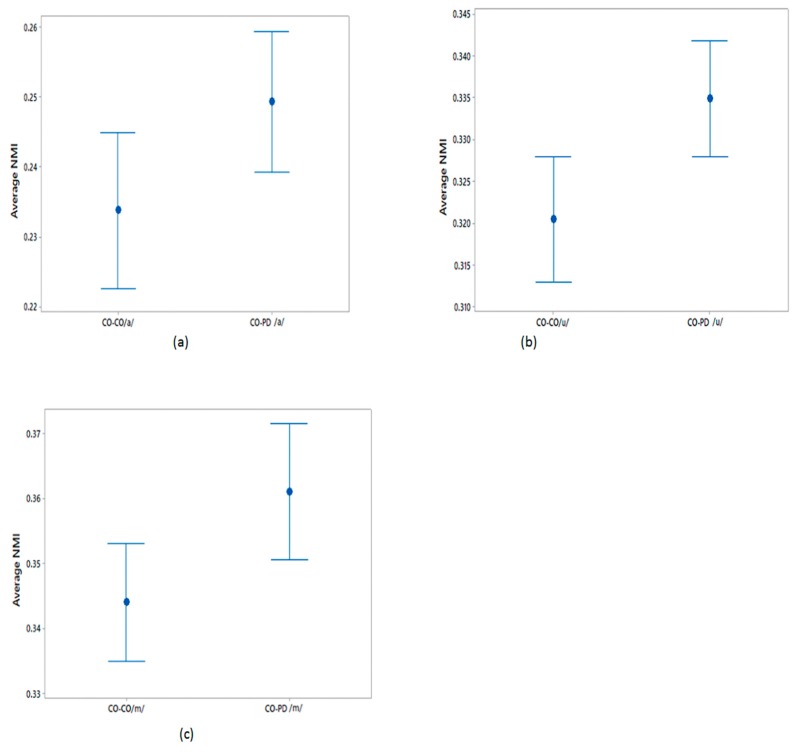
95% CI plots for the average NMI of CO–CO and CO–PD for (**a**) /a/; (**b**) /u/; (**c**) /m/.

The differences in the average NMI between CO–CO and CO–PD voices for each of the three phonemes were evaluated as described in Section 2.5. The average NMI values were compared using a one-way ANOVA test and the results are shown in Table 5. From this table, it can be seen that the sets of average NMI values for CO–CO and CO–PD were significantly different for all three phonemes. The plots of the 95% CI of the sets of average NMI values are shown in Figure 4 for within-group CO–CO and between-group CO–PD. Comparing the sets of average NMI values obtained from the different groups, for each of the phonemes there exists a notable difference in the average values, with only a moderate overlap between the groups.

#### 3.1.4. Comparative Speech Features

**Figure 5:**
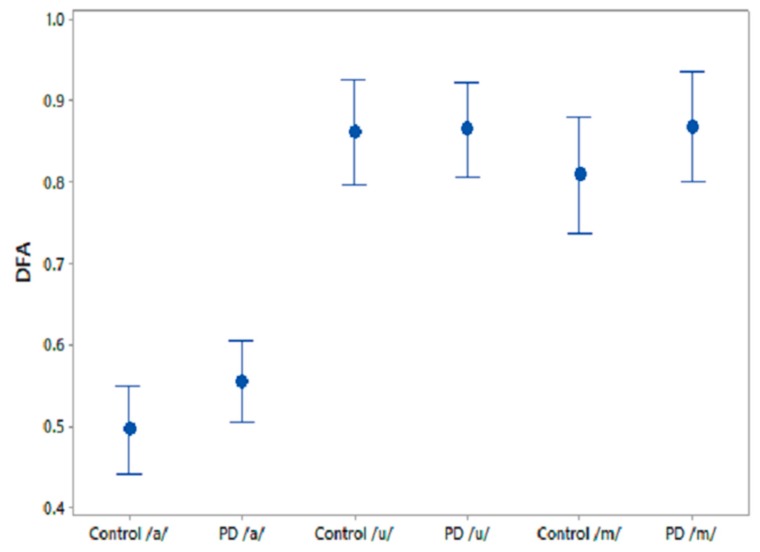
95% CI plots for the features DFA and FD for PD and CO.

A one-way ANOVA test was performed on the comparative speech features to test their efficiency at differentiating PD and CO. The results for the three phonemes are shown in Table 6. The feature, GNE, was significantly different between CO and PD for /u/ recordings and PPE norm showed similar trends for /m/ recordings. Figure 5 shows the 95% CI plots for the comparison speech feature DFA, which is associated with the self-similarity in speech signals. It is observed that DFA is higher for PD than for CO.

### 3.2. Classification Analysis

After verifying the two study hypotheses using statistical methods, the features FD and average NMI were employed in a binary classification problem with responses as PD and CO. The features were tested on a SVM classifier with linear kernel to differentiate PD and CO. The comparison features were also tested for the same binary classification problem using the SVM classifier. The performance of the features in the classifier was tested using a leave-one-subject-out validation method. The classification results for FD and average NMI are provided in Table 7 for each phonetic sound and the combination of phonetic sounds. The same has been reported for the comparison features in Table 8. Area under the curve (AUC), classification accuracy (CA), true negative/false negative (TN/FN) and false positive/true positive (FP/TP) have also been used to demonstrate the performance of the classification.

From these tables, it is observed that the classification based on FD and average NMI outperforms the other methods for the three phonemes and their combinations. It is also observed from Table 7 that the highest classification accuracy (0.81), with AUC 0.84, was obtained when the complexity features of the three phonetic recordings were combined. When comparing the individual classification accuracy from Table 7, it is observed that both the /u/ (0.78) and /m/ (0.78) complexity features showed equal efficiency at differentiating PD and CO, with AUC values of 0.84 and 0.83, respectively. While the classification accuracy was lower when using the features from the literature for the independent phonetic recordings (Table 8), the combination of their features resulted in an improvement in classification accuracy (0.67), with AUC 0.65 (Table 8).

## 4. Discussion

Human speech transmits information-rich signals [32] that are determined by habitual voice production combined with contextual factors relating to communication. In PD, depletion of dopamine in the basal ganglia causes the complex motor deficit of bradykinesia—reduced speed, reduced amplitude, loss of rhythm and loss of ability to sustain repetitive movement. Habitual actions such as speech and gait, which are associated with higher degrees of automaticity, are particularly affected [72,73]. It has been shown that over 80% of PD patients suffer from dysarthria [74,75]. This has been attributed to reduced respiratory power and to defects in the control of articulatory musculature [76]. Their voice becomes breathier, more monotonous and softer. The degradation of the voice production mechanism in PD patients leads to the generation of aperiodic and irregular voice characteristics, which has been identified with the help of acoustic analysis [56,74,75]. However, it is often difficult to observe these changes in the early stages of the disease with features extracted from phonation tasks [77,78].

This study has investigated the complexity in voices of PD patients using FD. We found a reduced complexity in phonetic sound recordings compared with CO subjects. Furthermore, from the computation of NMI we observed a significantly higher NMI within the PD group compared with the CO group. It was also found that CO–PD had higher NMI compared with CO–CO. The increase in the average NMI associated with PD can be explained by a reduced richness of voice. One explanation is that PD patients have less ability to modify their speech articulators. In comparison, there is a richness of content in healthy voices due to the ability of the person to continuously manipulate the spectrum of their speech, and to respond to auditory feedback. Thus, healthy people have a larger range of vocal parameters, which results in more variation between the signals. Hence, the lower observed proportion of shared information within the CO group. Effects of PD on the vocal apparatus [79] lead to a restricted voice production mechanism, with less richness than in healthy individuals [27]. PD voices can be considered to contain limited temporal and spectral information with respect to CO voices. This limitation in turn leads to the PD voices having higher similarity with other voices compared with CO–CO. Thus, the NMI of all voices, when paired with PD patients, would be increased. Lower NMI would indicate CO, while higher NMI would indicate PD. The threshold may be dependent on the local population.

The use of FD and NMI has shown higher AUC and more true positives. Another major advantage of using this method is the simplicity of the analysis. A number of speech features reported in other studies for the screening of PD [48,50,80] use measures of linear and nonlinear acoustic features that require the accurate detection of a fundamental frequency and reconstruction of the voice signal. FD and NMI do not require any such assessment and are more suited to automatic analysis. Another potential advantage, though not explored here, is that these measurements could, through training in a given population, overcome the confounding effects of accents and culture.

The novelty of this study is that it has found a reduction in FD in the voice of PD patients, showing the reduction in the richness of their speech and an increase in mutual information. It has also been shown that FD and NMI can differentiate PD speech from age-matched controls, even before clinically detectable speech impairment. This method could therefore be applied to early diagnosis as well as the clinical monitoring of PD patients. This study has also observed that /m/ is the best phoneme for differentiating between the two groups, followed by /u/, while /a/ shows the least sensitivity (Table 7). Another strength of our study is that it was able to show the PD vs. CO difference, without gender separation, thereby reducing the complexity of the potential applications of the method.

A study limitation is the relatively small number of participants. While this number is comparable to other similar studies, the sample is insufficient to evaluate differences caused by gender and ethnicity. Thus, while this method may be suitable for identifying people with early-stage dysarthria, it is not specific to the underlying disease. Another limitation of this work is that it has only been trialled for a single suburban cohort near Melbourne, Australia. To realise the full potential of this method, it is important to train and test this for different regions of the world, and for different group sizes.

## 5. Conclusions

This study has shown that the voice in PD has reduced complexity compared with CO. It has also shown that there is a potential for using NMI and FD of voice as a population screening tool for speech pathologies more generally. The proposed method does not require segmentation or identification of the fundamental frequencies, making it suitable for fast pathological speech analysis. We have also compared this approach to other acoustic features, and it was observed that NMI and FD outperform other features in differentiating between CO and PD. In this study, many PD patients did not exhibit dysarthria, suggesting that NMI and FD can be used to identify PD patients even when there is no noticeable change in their voices. The study has also shown that, of the three phonemes tested, /m/ was the most suitable for screening and /a/ was the least suitable. Another strength of this approach is that the differences between PD and CO were observed without needing to gender match.

## Figures and Tables

**Table 1:** Participant clinical information.

	PD Subjects	CO
Number of subjects	24	22
Age	71.83 ± 7.67	66.91 ± 6.22
UPDRS-III motor assessment	27.58 ± 2.58	2.64 ± 3.65
MoCA	27.58 ± 2.48	28.45 ± 1.37
Duration of disease in years	5.63 ± 3.00	-
Range of speech score in UPDRS	0–1	0

**Table 2:** FD compared between PD patients and CO.

Phonetic Sound	*f* Value	*p* Value
/a/	10.12	0.003 *
/u/	7.65	0.008 *
/m/	2.51	0.120

* denotes significance < 0.05.

**Table 3:** Range of average NMI values for CO–CO and PD–PD for three phonetic recordings.

Phonetic Sound	PD	CO
Average NMI		
/a/	0.212–0.304	0.192–0.276
/u/	0.293–0.361	0.284–0.358
/m/	0.291–0.414	0.289–0.384

**Table 4:** The differences between within-group NMI features for PD and CO using one-way ANOVA.

Feature	*f* Value	*p* Value
Average NMI /a/	10.18	0.003 *
Average NMI /u/	12.39	0.001 *
Average NMI /m/	12.38	0.001 *

* denotes significance < 0.05.

**Table 5:** Difference between CO–CO average NMI set and CO–PD off-state average NMI set using a one-way ANOVA test.

Phonetic Signal	*f* Value	*p* Value
Average NMI /a/	4.62	0.037 *
Average NMI /u/	8.58	0.005 *
Average NMI /m/	6.33	0.016 *

* denotes significance < 0.05.

**Table 6:** One-way ANOVA test of the features norm. PPE, GNE, DFA and ClQ between CO and PD off state.

Feature	*f* Value	*p* Value
Phoneme /a/		
Norm. PPE	0.017	0.898
GNE	2.765	0.103
ClQ	0.329	0.569
DFA	2.751	0.104
Phoneme /u/		
Norm. PPE	1.395	0.244
GNE	8.567	0.005 *
ClQ	0.003	0.957
DFA	0.004	0.949
Phoneme /m/		
Norm. PPE	4.807	0.033 *
GNE	3.321	0.075
ClQ	0.356	0.553
DFA	1.539	0.221

* denotes significance < 0.05.

**Table 7:** Classification results for the sustained phonetic sounds /a/, /u/ and /m/ using FD and NMI.

Task	AUC	CA	TN/FN	FP/TP
/a/	0.83	0.76	16/5	6/19
/u/	0.84	0.78	19/7	3/17
/m/	0.83	0.78	17/5	5/19
/a/ + /u/ + /m/	0.84	0.81	18/5	4/19

**Table 8:** Classification results for the sustained phonetic sounds /a/, /u/ and /m/ using four features from the literature.

Task	AUC	CA	TN/FN	FP/TP
/a/	0.17	0.46	10/13	12/11
/u/	0.35	0.46	8/11	14/13
/m/	0.73	0.65	12/6	10/18
/a/ + /u/ + /m/	0.65	0.67	15/8	7/16
